# Mast Cells: Key Contributors to Cardiac Fibrosis

**DOI:** 10.3390/ijms19010231

**Published:** 2018-01-12

**Authors:** Scott P. Levick, Alexander Widiapradja

**Affiliations:** Kolling Institute for Medical Research, University of Sydney, St Leonards 2065, Australia; alexander.widiapradja@sydney.edu.au

**Keywords:** heart, protease, tryptase, chymase, TNF-α, collagen, extracellular matrix, histamine

## Abstract

Historically, increased numbers of mast cells have been associated with fibrosis in numerous cardiac pathologies, implicating mast cells in the development of cardiac fibrosis. Subsequently, several approaches have been utilised to demonstrate a causal role for mast cells in animal models of cardiac fibrosis including mast cell stabilising compounds, rodents deficient in mast cells, and inhibition of the actions of mast cell-specific proteases such as chymase and tryptase. Whilst most evidence supports a pro-fibrotic role for mast cells, there is evidence that in some settings these cells can oppose fibrosis. A major gap in our current understanding of cardiac mast cell function is identification of the stimuli that activate these cells causing them to promote a pro-fibrotic environment. This review will present the evidence linking mast cells to cardiac fibrosis, as well as discuss the major questions that remain in understanding how mast cells contribute to cardiac fibrosis.

## 1. Introduction

Mast cells (MCs) are non-circulating immune cells that develop only once bone marrow-derived precursors have reached their target tissue. These tissue MCs then go through several stages of maturation driven primarily by the c-kit ligand, stem cell factor, with the final MC phenotype being highly dependent on the microenvironment in which they reside. There is now a substantial amount of evidence supporting a role for MCs in cardiac remodeling and heart failure [1]. In fact, MC numbers increase in the heart both in humans and in experimental animals in a wide variety of cardiac pathologies including myocardial infarction (MI) [2,3,4], hypertension [5,6,7], transplantation [8], myocarditis [9], volume overload [10,11], and in the failing heart [12]. Many of the effects of cardiac MCs involve regulation of the extracellular matrix (ECM), whether it be inducing ECM degradation or promoting increased ECM synthesis. The latter characterises cardiac fibrosis, which is a characteristic of almost all cardiac pathologies and is the focus of this review article. Cardiac MCs can influence fibrosis by direct effects on fibroblasts, as well as indirect effects, both brought about by the many proteases, cytokines, growth factors, and other products manufactured by these multi-faceted cells. This review article will discuss the evidence supporting a role for MCs in cardiac fibrosis by presenting studies that have utilised MC stabilizing compounds, rodents deficient in MCs, and specific inhibitors of MC proteases. This review will also discuss important unanswered questions in the field, including the elusive mediators that activate cardiac MCs causing them to promote fibrosis.

## 2. Studies Associating Mast Cells with Cardiac Fibrosis

MCs were first linked to cardiac fibrosis more than 50 years ago with the observation that these cells were increased in human hearts with endocardial fibrosis [13]. Since then, there have been numerous other observations that have associated MCs with cardiac fibrosis arising from multiple etiologies. In 1988, increased MCs were found to be associated with areas of fibrosis in biopsies obtained from 92 human diseased hearts by Turlington et al. [14]. In 1989, Olivetti et al. [6] observed an increased number of MCs in the right ventricle (RV) of rats following constriction of the pulmonary artery, a technique that results in RV fibrosis. Subsequently, Li et al. [8] reported that MCs were increased in human hearts following transplantation and that MC number correlated with fibrosis (*r* = 0.63). Strengthening this relationship was the observation that patients with high numbers of MCs at two weeks post-transplantation showed a 17% increase in fibrosis by week 3, whilst those patients with lesser numbers of MCs had only a 3.5% increase in fibrosis. Perhaps not surprisingly, patients in the high MC group also scored higher on the rejection scale.

Levels of the MC-specific amine, histamine, were reported to be elevated in experimental Chagas’ disease induced by infection of mice with Trypanosoma cruzi virus [15], with MCs in these mice appearing in areas of fibrosis [16]. Further, MC degranulation occurs soon after infection of mice with experimental myocarditis induced by coxsackievirus [17]. MC density also increases [18] in myocarditis and very strongly correlates with collagen volume fraction (*r* = 0.946) [19].

MCs were also linked to fibrosis in the hypertensive left ventricle (LV) when Panizo et al. [5] observed an increase in MC density in the LV of spontaneously hypertensive rats (SHR) that strongly correlated with collagen volume fraction (*r* = 0.87). Shiota et al. [7] also reported increased MC densities across the lifespan of the SHR. Even in stenotic aortic valves, MCs contained increased cathepsin G, which correlated with expression levels of collagen I and III [20]. More recently, Luitel et al. [21] confirmed in mice the earlier findings of Olivetti [6] in rats that MC density and degranulation increase in the RV following constriction of the pulmonary artery. Whilst these studies clearly show a strong association between MCs and fibrosis in the heart from varying etiologies, these associations do not establish causality. These studies are summarized in Table 1.

## 3. Evidence for the Causal Involvement of Mast Cells in Cardiac Fibrosis

### 3.1. Studies with Mast Cell Stabilizers

**Figure 1:**
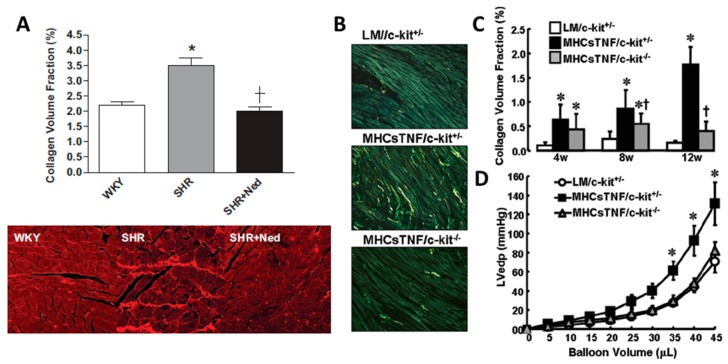
MC stabilization with nedocromil, or MC deficiency prevents cardiac fibrosis. (**A**) Quantification and representative picrosirius red-stained images (20× magnification) for left ventricle (LV) collagen volume fraction for Wistar Kyoto rats (WKY), spontaneously hypertensive rats (SHR), and SHR treated with the MC stabilizer, nedocromil (Ned, 30 mg/kg/day), * = *p* < 0.05 vs WKY, † = *p* < 0.05 vs SHR; (**B**) representative images of picrosirius red stained LV collagen in control mice (LM/c-kit^+/−^), TNF-α overexpressing mice (MHCsTNF/c-kit^+/−^), and TNF-α overexpressing mice crossed with MC-deficient mice (MHCsTNF/c-kit^−/−^); (**C**) quantification of collagen volume fraction in control LM//c-kit^+/−^ mice, MHCsTNF/c-kit^+/−^ mice, and MHCsTNF/c-kit^−/−^ mice; and (**D**) LV pressure-volume relationship for control LM//c-kit^+/−^ mice, MHCsTNF/c-kit^+/−^ mice, and MHCsTNF/c-kit^−/−^ mice. * = *p* < 0.05 vs LM/c-kit^+/−^, † = *p* < 0.05 vs MHCsTNF/c-kit^+/−^. (Copied with permission from Levick et al., Hypertension, 2009;53:1041–1047 (**A**); and Zhang et al., Circulation, 2011;124:2106–2116 (**B**–**D**)).

MC stabilizers prevent the release of MC mediators (e.g., histamine). This stabilization may ultimately involve blocking calcium channels, without which MC granules cannot fuse to the cell membrane and be exuded. Several studies have used this approach to examine the role of MCs in cardiac fibrosis. Palaniyandi et al. [19] demonstrated that the MC stabilizer disodium cromoglycate (also known as cromolyn) could dramatically reduce fibrosis in a model of myocarditis, whereby rats were injected with porcine cardiac myosin emulsified with complete freund’s adjuvant with *Mycobacterium tuberculosis* H37RA. A subsequent study confirmed the anti-fibrotic effect of cromolyn in myocarditis in rats [22]. We provided the first causal evidence that MCs play a role in cardiac fibrosis in the hypertensive heart [23]. SHR were treated with the MC stabilizing compound nedocromil (30 mg/kg/day) from 8 weeks of age (prior to the development of fibrosis) through to 24 weeks of age. This resulted in complete prevention of fibrosis in the LV, as determined by collagen volume fraction (Figure 1A). This included the observation that MC stabilization prevented macrophage recruitment and normalized cytokine profiles (IFN-γ, IL-4, IL-6 and IL-10). Interestingly, we found that IL-10 was dramatically decreased in untreated SHR, and was returned to normal after MC inhibition. In a previous study, Palaniyandi et al. [24] had demonstrated that IL-10 inhibited acute myocarditis-induced pathological changes in the heart, and that this likely involved the inhibition of MCs since histamine levels and MC density were reduced by IL-10. Thus, IL-10 may represent an endogenous MC inhibitor, with a loss of IL-10 leaving MCs susceptible to activation stimuli. Confirming the pro-fibrotic role of MCs in the pressure overloaded heart, Kanellakis et al. [25] showed that cromolyn prevented LV fibrosis in mice with transaortic constriction. Similarly in the atria, the MC stabilizer, cromolyn, prevented fibrosis following transaortic constriction-induced pressure overload on the heart [26]. Even in STZ-induced diabetic hearts, nedocromil was able to reduce cardiac fibrosis [27]. More recently, Li et al. [28] found that nedocromil (30 mg/kg/day) prevented fibrosis from developing in rats following five weeks of transaortic constriction. Thus, the MC stabilzer studies strongly argue for a role for MCs in cardiac fibrosis. However, one must be aware of possible off target effects of these compounds, such as inhibition of sensory nerves. These studies are summarized in Table 2.

### 3.2. Studies with Mast Cell-Deficient Rodents

#### 3.2.1. Types of Mast Cell-Deficient Rodents

There are several mutant mice that have been utilized as models of MC-deficiency to study MC function in vivo including *Kit^W/W-v^* and *Kit^W/W-sh^* mice [38,39,40,41]. These mouse strains carry spontaneous loss of function mutations at both alleles of the dominant *white spotting* locus (*W*, i.e., *c-kit*). This mutation exhibits a significant reduction in c-kit tyrosine kinase-dependent signalling, hence disrupting normal MC development and survival [39,42]. The different mutant alleles of c-kit reflect the variable non-MC-related effects that these mice display. The mutated *W* allele gives rise to truncated c-kit without the transmembrane domain, resulting in no protein expression on the cell surface [43]. Alternatively, the *W^v^* mouse has a point mutation at the tyrosine kinase-encoding domain of c-kit. Unlike the *W* mouse, *Kit^W/W-v^* mice still express the c-kit protein on the cell surface, although with reduced tyrosine kinase activity [43,44]. *Kit^W/W-v^* mice have no detectable MCs in multiple organs by the time they reach 6 to 8 weeks of age [39]. However, due to malfunction of the c-kit protein, these mice display phenotypic abnormalities such as macrocytic anaemia, infertility, impaired melanogenesis, lack of intestinal cells of Cajal, spontaneous dermatitis, gastric ulcers and duodenum dilatation [45,46,47,48,49,50,51,52,53]. This strain has traditionally been the most popular strain used to study MC-deficiency.

The *W-sash* (*Kit^W/W-sh^*) mutation is an inversion mutation in the transcriptional regulatory elements, upstream of the c-kit transcription start site of mouse chromosome 5, resulting in functionally impaired c-kit protein [54]. This mutation was first described 23 years ago from crossing two inbred strain mice (C3H/HEH × 101/H), although it is only fairly recently that this strain has gained popularity as a model of MC-deficiency in vivo [40,55]. Adult *Kit^W/W-sh^* mice are MC-deficient at multiple anatomical sites [56], however, unlike the *Kit^W/W-v^* mouse, they are fertile and not anaemic [40,57]. They also exhibit normal levels of myeloid cells, B cells, T cells, dendritic cells, and basophils [58]. Importantly, like the *Kit^W/W-v^* mouse, *Kit^W/W-sh^* mice can be successfully reconstituted with bone marrow derived mast cells with normal c-kit expression by adoptive transfer via intraperitoneal, intradermal or intravenous injection [55,56,58]. A comprehensive study by Grimbaldeston et al. [58] details the advantageous of *Kit^W/W-sh^* mice over *Kit^W/W-v^* mice.

Another mutant mouse that can be used to study MC biology is the *steel-Dickie (Sl^d^)* mouse. This mutation occurs due to a 4.0 kilobase intragenic deletion and truncates the *Sl* coding sequence. Mast cell growth factor (MGF) is encoded by the *Sl* gene, hence this mutation results in soluble truncated growth factor that lacks both transmembrane and cytosplasmic domains [59]. *Sl^d^* mice carry a homozygous mutation in the *Sl* gene as a complete deletion of *Sl* alleles, resulting in the complete absence of MGF and is lethal [60,61,62]. Phenotypically, *Sl^d^* mice exhibit melanocytes defects, severe anaemia and sterility [63].

There is also a MC-deficient rat. The *Ws/Ws* rat has a 12-base deletion in the tyrosine kinase domain of c-kit, the receptor for stem cell factor. The *Ws/Ws* phenotype is otherwise normal except for white spotting of the skin and macrocytic anemia that is spontaneously ameliorated by 10 weeks of age [41]. Therefore, these rats do not exhibit the severe anemia seen in some MC-deficient mouse strains.

#### 3.2.2. Pro-Fibrotic Role for Mast Cells

Hara et al. [29] were the first to use MC-deficient mice to evaluate cardiac fibrosis in any form. They used male *Kit^W/Wv^* mice exposed to abdominal aortic banding for 15 weeks and found that whilst perivascular fibrosis occurred in WT mice with banding, collagen levels were normal in *Kit^W/Wv^* mice. To definitively confirm the role of MCs in MC-deficient mice, MCs must be replaced by adoptive transfer. Unfortunately, Hara et al. were unsuccessful in their attempt to replenish MCs in *Kit^W/Wv^* mice, thus, could not confirm that a lack of MCs was solely responsible for the resistance to adverse remodelling. The inability to reconstitute MCs to the hearts of these mice may have been due to beginning reconstitution just 2 days before initiating banding. Typically, it takes 6 to 8 weeks for injected MCs to reconstitute to the heart. Liao et al. [26] used the *Kit^W/Wv^* mouse to investigate the contribution of MCs to atrial fibrosis following transaortic constriction. These authors reported that mice deficient in MCs were protected against atrial fibrosis, however, they also did not perform MC reconstitution experiments. In an interesting study, Zhang et al. [30] investigated the role of MCs in cardiac fibrosis in a model of TNF-α overexpression. Mice with cardiac-restricted TNF-α overexpression were crossed with *Kit^W/W-sh^* mice. While fibrosis developed in the hearts of TNF-α overexpressing mice, this did not occur in MC-deficient TNF-α overexpressing hearts, indicating that MCs mediate the pro-fibrotic actions of TNF-α in this setting (Figure 1B,C). Further, the lack of MCs restored normal diastolic function as indicated by normalisation of the LV pressure volume relationship (Figure 1D). This study shows that in the setting of elevated TNF-α, MCs mediate the pro-fibrotic actions of TNF-α and that MC-mediated cardiac fibrosis contributes to diastolic dysfunction. This study offers probably the most conclusive evidence to date that MCs contribute to cardiac fibrosis, however, the caveat here is that this is an artificial up-regulation of TNF-α, which may or may not be relevant to conditions such as hypertension. These studies are summarized in Table 2.

#### 3.2.3. Anti-Fibrotic Role of Mast Cells

Despite the evidence that MCs are pro-fibrotic in the heart, there does appear to be some exceptions. Martin Hauer-Jensen’s laboratory has used the *Ws/Ws* MC-deficient rat to identify some of these exceptions. In one study, Joseph et al. [31] fed *Ws/Ws* rats a diet high in homocysteine for 10 weeks to induce hyperhomocysteinemia that causes perivascular and interstitial cardiac fibrosis, without concomitant cardiomyocyte hypertrophy. Both forms of fibrosis were further increased in *Ws/Ws* rats fed homocysteine, indicating that MCs were protective in this setting. In another study on cardiac injury caused by radiation (single dose at 18 Gy), Boerma et al. [32] found that MC-competent rats had increased collagen III compared to rats deficient in MCs. However, this was not the case for collagen I. These studies are summarized in Table 2.

The reasons why MCs appear to be pro-fibrotic except in the instances of hyperhomocysteinemia and radiation are not clear. One tempting explanation is the use of MC-deficient rats in the studies indicating an anti-fibrotic role versus MC-deficient mice in the studies supporting a pro-fibrotic role. However, MC stabilisers in rats appear to be anti-fibrotic, arguing against this hypothesis. It may be that MCs are directed to take on a pro-fibrotic phenotype when the stimulus involves altered cardiovascular hemodynamics (e.g., hypertension) or infection (e.g., myocarditis), whereas remodelling not related to these types of stimuli invoke an anti-fibrotic MC phenotype. Deeper analysis of the types of mediators released by MCs in each of these settings will be required to confirm or refute this hypothesis.

## 4. MC products

**Figure 2:**
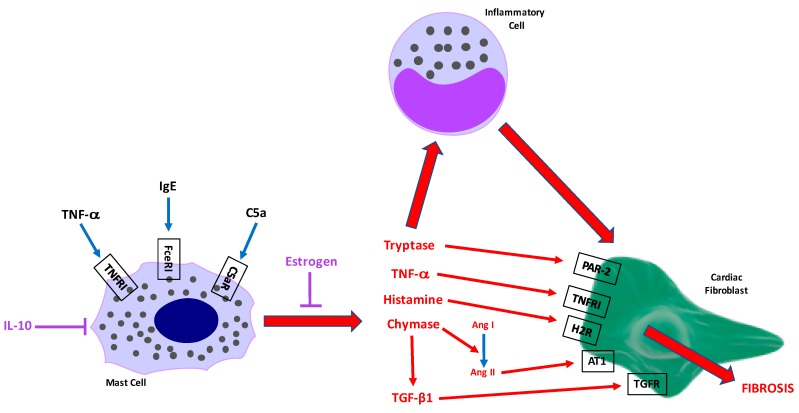
Schematic depicting potential MC activation stimuli and interactions with other cell types that lead to cardiac fibrosis. Candidates for cardiac MC activation include IgE, TNF-α, and C5a. These then cause the release of MC mediators including the proteases tryptase and chymase, TNF-α, histamine, and TGF-β1. These mediators can then have direct effects on cardiac fibroblasts, but may also contribute to an inflammatory response that then activates cardiac fibroblasts via numerous cytokines. IL-10 and estrogen likely oppose cardiac MC activation/degranulation.

Figure 2 indicates specific mediators released by MCs and their potential contributions to cardiac fibrosis. These mediators are discussed below.

### 4.1. Proteases

**Figure 3:**
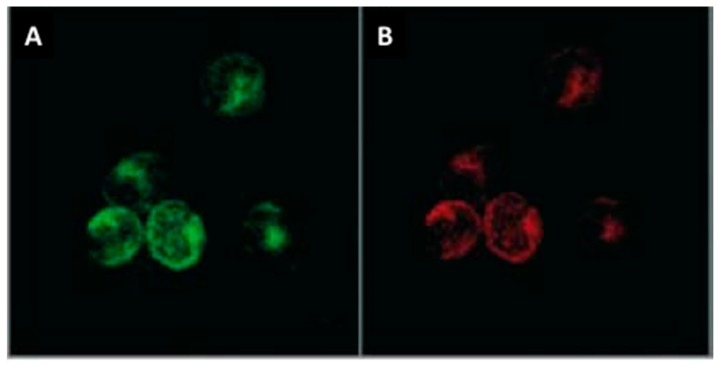
Immunolabelling of chymase (**A**, green) and tryptase (**B**, red) in cardiac mast cells isolated from rats (400× magnification; Copied with permission from Morgan et al., Inflamm Res, 2008;57:1–6).

Cardiac MC phenotype has not been well studied. However, cardiac MCs, like MCs in general, store large amounts of specific proteases. Cardiac MCs fall under the connective tissue type MC phenotype since they contain both chymase and tryptase (Figure 3). On the other hand, mucosal MCs are tryptase^+^, but chymase^-^. Most of the work pertaining to cardiac MC products that contribute to fibrosis has focused on these proteases.

#### 4.1.1. Chymase

Chymase is a chymotrypsin-like serine protease stored in the granules of MCs. In a mouse model of transaortic constriction, Hara et al. [29] observed an increase in *Mcp5* in the heart, one of the mouse genes for chymase. Chymase activity in the heart was reported to increase 5.2-fold in hamsters with hypertension induced by the two-kidney, one-clip approach [64]. Similarly, there is evidence of chymase up-regulation in humans, with chymase mRNA and protein increased in patients with aortic stenosis undergoing valve replacement surgery [65].

**Figure 4:**
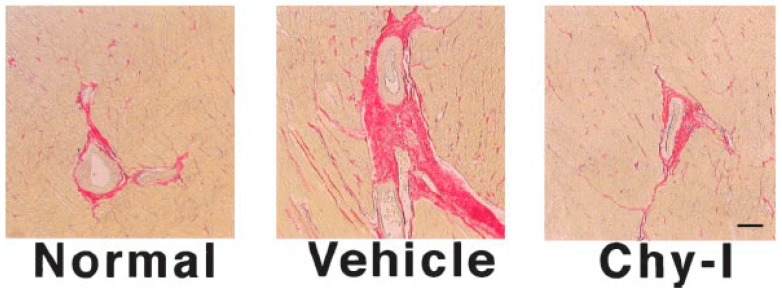
Picrosirius red-stained images of perivascular fibrosis in normal, paced (vehicle), and paced plus the chymase inhibitor SUNC8257 (Chy-I) dog hearts (100× magnification; Copied with permission from Matsumoto et al., Circulation, 2003;107:2555–2558).

The first study to demonstrate a causal role for chymase in cardiac fibrosis was by Matsumoto et al. [33]. In this study, heart failure was induced by rapid pacing in beagles (270 bpm, 22 days). Dogs with heart failure were treated with the chymase inhibitor SUNC8257 (10 mg/kg, orally twice a day), with chymase inhibition reducing collagen I and III mRNA levels and fibrosis as determined by picrosirius red staining (Figure 4). In the setting of MI-induced remodelling in rats, the chymase inhibitor, NK3201 reduced collagen I and III levels as well as fibrosis following 4 weeks post-MI, although it was unclear whether this analysis only included the infarct region or the entire LV [34]. Intriguingly, whilst there was a small improvement in diastolic function with chymase inhibition, as determined by E/A ratio, LV dilatation and systolic function were not improved. In an interesting study, Matsumoto et al. [35] investigated the role of chymase in cardiac remodelling caused by intermittent hypoxia mimicking sleep apnoea. Mice were placed in chambers that delivered intermittent hypoxia (30 s of 4.5% to 5.5% O_2_ followed by 30 s of 21% O_2_ for 8 h/day during the daytime) or normoxic conditions for 10 days. In addition to other remodelling parameters, perivascular fibrosis was increased by hypoxia and reduced by the chymase inhibitor, NK3201.

In the Matsumoto study in dogs, TGF-β1 mRNA was reduced by chymase inhibition whereas ACE mRNA was not. Further, Shimizu et al. [66] observed that aortic banding in male Syrian hamsters induced an increase in chymase activity and a decrease in angiotensin converting enzyme (ACE) activity, while causing cardiac fibrosis. The authors concluded that this indicated that chymase was primarily responsible for angiotensin II formation in this setting. These studies reflect the two known mechanisms likely to underlie chymases role in promoting fibrosis (i.e., angiotensin II and TGF-β1). Evidence has shown that chymase plays a role in the formation of angiotensin II in a non-canonical pathway of the renin angiotensin system (RAS). Chymase acted as an angiotensin-(1–12) converting enzyme in generating angiotensin II in SHR neonatal myocytes [67]. Chymase was also found to be responsible for this bio-conversion in the left atria of patients undergoing surgery for treatment of resistant atrial fibrillation or LV of normal patients dying from motor vehicle accidents [68,69]. Further supporting this, cardiac angiotensin II formation was reduced by the chymase inhibitor NK3201 in a mouse model of intermittent hypoxia [35]. Thus, one mechanism by which chymase exerts pro-fibrotic actions is via angiotensin II. Although, one needs to be aware of species differences in the contribution of chymase versus ACE to the cardiac angiotensin II pool. Balcells et al. [70] have noted that the human heart has dramatically more angiotensin II than other species, with dog being second, followed by mouse, rabbit, and rat. Human cardiac angiotensin II was almost entirely accounted for by chymase activity, as was also the case in the dog [70]. Approximately 25% of cardiac angiotensin II formation in the rat was attributed to chymase, whereas the contribution was less again in the rabbit and mouse. Akasu et al. [71] found similar results. They reported that angiotensin II was almost entirely accounted for by chymase in human, hamster, dog, and marmoset hearts. Pig and rabbit hearts showed ACE as the primary mechanism for angiotensin II synthesis. Interestingly, and in contrast to Balcells et al. [70], Akasu et al. [71] found that chymase was the primary determinant of angiotensin II formation in the rat heart. The mouse heart was not investigated.

The other pathway by which chymase exerts pro-fibrotic actions involves TGF-β1. At a cellular level, treatment of isolated neonatal cardiac fibroblasts with chymase (15–30 ng/mL) for 24 h resulted in cell proliferation [72]. This was accompanied by increased mRNA and protein levels of TGF-β1. Chymase up-regulation of TGF-β1 was independent of angiotensin II since blockade of AT_1_ and AT_2_ receptors did not alter TGF-β1 production. The proliferative and collagen producing effects of chymase could be reduced by a neutralising antibody to TGF-β1. The downstream mediator of TGF-β1 was Smad2/3 and not p38 or ERK pathways. In vivo, cromolyn reduced TGF-β1 levels in rats with myocarditis [19]. Shiota et al. [7] demonstrated that activated MCs were a major source of TGF-β1 and were localised to areas of fibrosis in 12 month and 20 month old SHR that were in advanced stages of LV hypertrophy and heart failure, respectively. In a mouse model of TNF-α overexpression, crossing these mice with MC-deficient mice reduced TGF-β1 levels as well TGF-β receptors in the heart [30].

#### 4.1.2. Tryptase

Tryptase is a serine protease also stored in the granules of MCs. Fewer efforts have focused on the role of tryptase in cardiac fibrosis, even though tryptase levels increase in fibrotic hearts [23,28]. Mice with encephalomyocarditis virus-induced myocarditis show up-regulated mRNA levels of *Mcp6*, the gene for tryptase, 14 days after infection, which tracked with an increase in collagen I gene expression [18]. Using an in vitro approach, we demonstrated that tryptase could increase ECM synthesis by cardiac fibroblasts after 72 h [23]. Interestingly, proliferation was induced much more rapidly (24 h), suggesting that a fibroblast proliferative response might be the primary action of tryptase. In a follow-up study, we demonstrated that the effects of tryptase on cardiac fibroblasts were mediated by protease activated receptor-2 (PAR-2), which induced selective MAP kinase pathways with ERK1/2 mediating the pro-fibrotic actions of tryptase on cardiac fibroblasts, with no involvement from p38 or JNK [36]. This pathway also mediated cardiac fibroblast conversion to the myofibroblast phenotype. Critically, blockade of PAR-2 with FSLLRY (10 μg/kg/day) in SHR prevented fibrosis from occurring, independent of blood pressure. Thus, the role of tryptase appears to be more direct than is the case for chymase. In another follow-up study, we identified an autocrine/paracrine response by cardiac MCs mediating their own protease release. Inhibition of tryptase with nafamostat mesilate (5 mg/kg/day) reduced plasma chymase levels in rats with transaortic constriction [28]. To investigate this further, sections of rat LV were cultured in a novel tissue culture system and treated with tryptase. Tryptase caused the release of chymase into the media and a concomitant increase in collagen production that could be reduced by the chymase inhibitor chymostatin. These results suggest that tryptase also acts in an autocrine/paracrine manner to induce chymase release from MCs, and subsequent fibrosis.

### 4.2. Other Mast Cell Products

MCs release many products other than proteases that are capable of influencing the ECM. Several of these will be discussed below. However, it is important to recognize that many of these products can be produced by other cell types, and the relative contribution of the MCs to the overall pool of some of these products is unclear.

#### 4.2.1. Histamine

The human heart contains considerable amounts of histamine (1035 ± 65 ng/g of atrial tissue) [73]. Histamine is the classic MC product mediating hypersensitivity reactions, and this is also true in the heart, which participates in anaphylaxis. However, histamine can also contribute to cardiac remodelling [74]. In the most direct assessment to date, Zeng et al. [37] performed transaortic constriction on H2 histamine receptor deficient mice (H2R^−/−^, Table 2). After four weeks, H2R^−/−^ mice showed reduced cardiac fibrosis and slightly improved systolic function, indicating a role for histamine in cardiac fibrosis. The investigators performed additional studies in isolated cardiac fibroblasts and determined that both histamine and the H2R-selective agonist amthamine dihydrobromide increased protein levels of calcineurin; this was prevented by the H2R antagonist famotidine. Importantly, H2R activation also up-regulated myofibroblast conversion, fibronectin production, and procollagen I and III up-regulation at the gene level. Calcineurin was subsequently shown to mediate fibroblast proliferation, fibronectin production, and collagen gene regulation in response to H2R activation. This study clearly shows the capability of histamine to have direct effects on cardiac fibroblasts. Interestingly though, the actions of histamine may extend beyond this. Of the four known histamine receptors, three (H1, H2 and H3) are found in the heart. Due to their localisation, the actions of these receptors in the heart are extremely complex. H1R and H2R are present in the sinoatrial and atrioventricular nodes of the heart, suggesting regulation of heart rate [75]. H1R modulates cardiac autonomic nerve function [76,77]. Interestingly, histamine enables noradrenaline release in the rat heart via H2R [78]. Given the pro-fibrotic properties of noradrenaline, this raises the possibility that MC-mediated release of noradrenaline via H2R could promote fibrosis. In fact, a recent clinical study linked H2R antagonist use to a 62% reduced risk of heart failure [79]. Cardiac sensory nerves possess H3R. Our recent findings that the sensory nerve neuropeptide substance P plays a critical role in cardiac fibrosis in the hypertensive heart raises the possibility that MC histamine could be responsible for its release [80]. If this is the case it sets up an interesting feed forward mechanism since we have data indicating that substance P and its cognate receptor the neurokinin-1 receptor are responsible for the increase in MC density observed in the hypertensive heart, but neurokinin-1 receptors do not contribute to MC activation in this setting. Thus, other stimuli activate MCs, potentially resulting in histamine release and amplification of the substance P response.

#### 4.2.2. Components of the Renin Angiotensin System

Renin is the first enzyme in the RAS, its role being to cleave angiotensinogen to angiotensin I, which in turn can be cleaved by ACE or chymase to active angiotensin II. In 2004, Roberto Levi’s group demonstrated that rat cardiac MCs contain renin [81]. Extrapolating the relevance of this finding to humans, Levi’s group also showed that the human MC line HMC-1 produced active renin that could convert angiotensinogen to angiotensin I. Subsequently, Hara et al. [82] confirmed the presence of renin mRNA in HMC-1 cells and further identified angiotensinogen mRNA in these cells. Interestingly, these authors identified pre-formed angiotensin II in HMC-1 cells that was released in response to calcitonin gene-related peptide. Although MC-derived renin contributes to ischaemia-induced arrhythmias in the heart [83], no specific evidence shows directly the role of MC-derived components of the RAS in cardiac fibrosis. However, given the known pro-fibrotic effect of angiotensin II, it is reasonable to assume that MC RAS contributes at least to some degree to cardiac fibrosis.

#### 4.2.3. TNF-α

Immunolabelling indicates that MCs are likely the main source of TNF-α in the heart [84,85]. MC stabilizers such as ketotifen and cromoglycate prevented TNF-α release in hearts undergoing ischemia reperfusion, further supporting this supposition [86]. MC-derived TNF-α has been shown to stimulate collagen production by dermal fibroblasts [87], however, the extent to which MC-derived TNF-α contributes to cardiac fibrosis has not yet been investigated.

#### 4.2.4. TGF-β

The role of MCs in generating TGF-β via chymase has already been discussed in Section 4.1.1, however, direct production of TGF-β by MCs could also contribute to organ fibrosis. Inhibition of TGF-β1 has been shown to mediate the pro-fibrotic effects of MCs on dermal fibroblasts in culture [87]. Although this suggests effects of MC-derived TGF-β, the alternate possibility cannot be ruled out that chymase induced activation of TGF-β produced by fibroblasts. There are no studies directly investigating the contribution of MC-derived TGF-β to cardiac fibrosis.

#### 4.2.5. Matrix Metalloproteinases

Investigation of MCs in airways of dogs found that these cells produce matrix metalloproteinase (MMP)-2 and -9 (gelatinase A and B) [88]. Stem cell factor selectively up-regulated MMP-9 [88]. Interestingly, TGF-β opposed the actions of stem cell factor on MMP-9. The significance of MMPs is their ability to regulate the ECM, whether it be by initiating degradation, or alternatively, inducing pro-fibrotic responses [89]. While MC regulation of MMPs has been established in cardiac volume overload [10,90], this results in ECM degradation in that setting. The contribution of MCs to MMPs has not been investigated in relation to cardiac fibrosis. However, conditioned media from MCs was able to increase MMP-2 activation in neonatal cardiac fibroblasts [91]. Additionally, chymase inhibition prevented MMP-9 activation in pigs undergoing ischemia reperfusion [92].

## 5. Important Questions

While there is accumulating evidence that MCs contribute to cardiac fibrosis, there are several important questions that remain unanswered.

### 5.1. What Activates Cardiac Mast Cells?

Probably the most pressing question is what are the stimuli that activate these cells causing them to promote fibrosis? There are several good candidates still to be investigated. These are depicted in Figure 2.

#### 5.1.1. Immunoglobulin E

Like all MCs, cardiac MCs possess the IgE receptor, FcεRI. This is evidenced by their degranulation in response to antibody against FcεRI in vitro [93]. Interestingly, FcεRI activation causes release of histamine and tryptase, as well as leukotriene C_4_ and prostaglandin D_2_ by isolated human cardiac MCs [94]. As discussed earlier, MC histamine and tryptase are linked to cardiac fibrosis. Surprisingly, the role of IgE and FcεRI in causing cardiac fibrosis has not yet been investigated.

#### 5.1.2. TNF-α

TNF-α receptor I (TNFRI) and TNF-α receptor II (TNFRII) mediate the actions of TNF-α. *TnfrI^−/−^* mice have improved cardiac remodelling responses (including fibrosis) following MI, while *TnfrII^−/−^* mice have worse fibrosis. Thus, TNFRI exacerbates remodelling leading to heart failure, whereas TNFRII has cardioprotective actions. The evidence implicating TNF-α and TNFRI comes from studies that crossed TNF-α overexpressing mice with MC-deficient mice. TNF-α overexpressing mice develop fibrosis; this fibrosis is reduced in mice lacking MCs. This indicates that MCs mediate the pro-fibrotic effects of TNF-α, implying that TNF-α plays a role in activating MCs. What is not clear from that study is whether this is direct activation of MCs by TNF-α via TNFRI or whether the activation is indirect with TNF-α up-regulating other mediators that then activate cardiac MCs. This question requires MC-specific deletion of TNFRI. Also, whether MC-derived TNF-α represents a viable treatment target is questionable given the failure of targeting TNF-α in heart failure patients.

#### 5.1.3. Complement 5a

Patella et al., demonstrated more than 20 years ago that isolated human cardiac MCs degranulate in response to the complement factor, C5a [93]. The response to C5a was more rapid than to IgE and reached the same maximal response as IgE. However, Füreder et al. [95] subsequently reported that only 5% to 15% of human cardiac MCs that they examined possessed the C5a receptor and that human cardiac MCs did not release histamine in response to C5a. Nevertheless, coronary infusion of C5a (500 ng) in pigs undergoing MI led to an increase in coronary histamine levels indicating MC activation by C5a [96]. It will require MC-specific deletion of the C5a receptor to help resolve this question.

### 5.2. What Are the Specific Mechanisms by Which Mast Cells Cause Cardiac Fibrosis?

Despite clear evidence from multiple animal models that MCs are involved in cardiac fibrosis and that MC proteases mediate these actions along with possible contributions from other MC mediators, we know very little about the specifics. One example is the temporal involvement of MCs in cardiac fibrosis. Our own belief is that these cells are important in initiating fibrosis, but may not be involved continually throughout the process. This somewhat stems from our experience in MC regulation of the ECM in volume overload models where MC density increases in the first few days following initiation of volume overload, before returning to normal [90]. This suggests that MCs are important early to initiate processes that then continue throughout the remodelling process. Supporting this, MCs density is increased in the young SHR [7] and continued to be increased at 8 and 12 weeks of age, when fibrosis develops, before returning to normal levels by 16 weeks of age once fibrosis is established. Interestingly, MC density increases again around the age that heart failure develops in the SHR [7].

**Figure 5:**
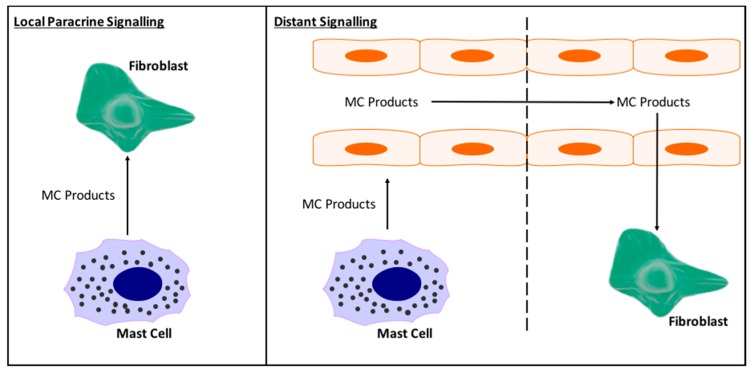
Schematic depicting the possible interactions between MCs and fibroblasts in the heart. (**Left**) MCs may act in a paracrine manner to signal only to fibroblasts in their local area. This would limit direct MC-fibroblast interactions as a mechanism by which MCs cause fibrosis; (**Right**) Alternatively, MC products may be taken up in the general coronary circulation allowing their products to be distributed to fibroblasts throughout the heart. This would allow for greater MC-fibroblast interactions.

Also undetermined are the specifics of MC interactions with cardiac fibroblasts. In vitro it has been shown that MCs can act directly on cardiac fibroblasts to induce myofibroblast conversion, proliferation, and excess collagen synthesis [36,72], however, do these direct interactions actually occur in vivo, and to what extent? While cardiac MC number does vary between species, overall there are very few MCs in the heart. Clearly, although few in number they have a significant impact, however, it does raise the question of how many cardiac fibroblasts and MCs can actually interact in a paracrine manner in vivo. This we do not know. We do not even know whether released MC products circulate in the heart, giving them the opportunity to reach fibroblasts from remote parts of the heart, or whether they just act locally. Figure 5 depicts local versus distant interactions between MCs and cardiac fibroblasts.

Also likely important in MC modulation of cardiac fibrosis are interactions with other inflammatory cells (Figure 2), especially if direct MC-fibroblast interactions are minimal. Conditioned media from bone marrow-derived MCs activated with the calcium ionophore A23187 (500 ng/mL) dramatically up-regulated VCAM-1, ICAM-1, P-selectin, and E-selectin in mouse heart endothelial cells, suggesting a role for MCs in inflammatory cell recruitment [97]. In support of this, we had observed reduced numbers of macrophages in the SHR LV following treatment with nedocromil compared to untreated SHR [23]. This qualitative assessment suggests a role for MCs in the recruitment of macrophages to the fibrotic heart, however, quantitative assessment of this effect is still lacking. Should MCs regulate macrophage recruitment, then it should be determined as to whether this is a direct effect on macrophages, or whether other cells are involved (i.e., MC activation of endothelial cells). We also reported that MC stabilization reduced IFN-γ and IL-4 production, demonstrating the contribution of MCs to the overall cytokine pool, whether directly or indirectly.

Very little work has investigated MC-cardiomyocyte interactions. In our own studies, we did not observe any significant effect on cardiac hypertrophy, either by MC stabilization with nedocromil or inhibition of tryptase with FSLLRY [23,36]. Chymase appears to be able to induce cardiomyocyte death by entering these cells and inducing translocation of nuclear receptor subfamily 4A1 (NR4A1) from the nucleus to the cytoplasm. This was identified in ischemia reperfusion injury; the significance of this to cardiac fibrosis has not been explored. Media from cultured MCs can invoke death in cultured neonatal cardiomyocytes, with a neutralizing antibody against chymase opposing this effect [98]. Cardiomyocyte death is a stimulus for cardiac fibrosis. Thus, MC interactions with cardiomyocytes could be a stimulus for fibrosis, however, this is yet to be established.

### 5.3. What Is the Cardiac Mast Cell Phenotype and Are There Gender Differences?

A large amount of the work contributing to our understanding of the role of MCs in adverse cardiac remodeling has come from the laboratory of Joseph Janicki. Recently, the Janicki lab produced evidence indicating that differences in MC phenotype may underlie cardioprotection in pre-menopausal females. Intact and ovariectomised (ovx) female rats were exposed to pressure overload induced by transaortic constriction. LV MC density did not increase in intact females, but did increase in oxv rats [99]. Further, whilst there was a small increase in LV chymase levels in intact rats, the increase was greater in ovx animals. This was also the case for TGF-β1, presumably related to the increase in chymase. When oxv female rats were treated with estrogen, MC density, LV chymase, and TGF-β1 were reduced, as was fibrosis. This suggests that estrogen in pre-menopausal females provides a level of protection from cardiac fibrosis by reducing the ability of MCs to either: (1) respond to activation stimuli and therefore reduce levels of MC proteases that contribute to fibrosis in males; or (2) reduce production of mediators responsible for promoting fibrosis. Essentially, phenotypic differences exist between male and female MCs and this may underlie pre-menopausal cardioprotection in females (Figure 2). This is an extremely interesting concept that needs to be further explored.

These potential phenotypic differences between male and female MCs leads to another gap in our cardiac MC understanding; very little is known about the cardiac MC phenotype in general. This is an area that has been badly neglected. We know that cardiac MCs are tryptase^+^/chymase^+^ (Figure 3), as well as containing histamine and TNF-α [84,94,100]. Patella et al. [94] have demonstrated that isolated human cardiac MCs produce leukotrienes in response to activation by C5a. Beyond these few mediators, essentially nothing is known about cardiac MC phenotype. Whether cardiac MC sub-populations exist, as is the case for macrophages and T cells, is currently unknown, but should become a focus of investigation.

## 6. Conclusions

There is strong experimental evidence that MCs contribute to cardiac fibrosis, at least in part through the release of MC-specific proteases. However, the specifics of how MCs promote fibrosis is not clear, including the role of non-protease MC products, the stimuli that activate cardiac MCs, how cardiac MCs and fibroblasts interact in vivo, and specifics of cardiac MC phenotype (e.g., sub-populations and gender differences). These questions must be answered if targeting MCs is to eventually become a therapeutic approach in humans.

## Figures and Tables

**Table 1:** Summary of in vivo studies associating mast cells (MCs) with cardiac fibrosis.

Species	Model/Pathology	Outcome	Heart Chamber	References
Human	Fibrosis	↑ MC	LV	[13,14]
Rat	Pulmonary hypertension	↑ MC	RV	[6]
Human	Transplantation	MC number correlated with fibrosis	LV	[8]
Mouse	Myocarditis	↑ histamine, ↑ MC correlated with fibrosis	LV	[15,19]
Rat	Hypertension	↑ MC correlated with fibrosis	LV	[5]
Mouse	Pulmonary hypertension	↑ MC	RV	[21]

LV = Left ventricle, RV = Right ventricle.

**Table 2:** In vivo studies establishing cause and effect between MCs and cardiac fibrosis.

Species	Intervention	Pathology	Outcome	Heart Chamber	Reference
MC Stabilizers					
Rat	Cromolyn	Myocarditis	↓ fibrosis	LV	[19,22]
Rat	Nedocromil	Hypertension	↓ fibrosis	LV	[23]
Mouse	Cromolyn	Transaortic constriction	↓ fibrosis	LV	[25]
Mouse	Cromolyn	Transaortic constriction	↓ fibrosis	Atria	[26]
Mouse	Nedocromil	STZ-induced diabetes	↓ fibrosis	LV	[27]
Rat	Nedocromil	Transaortic constriction	↓ fibrosis	LV	[28]
MC-deficient Rodents					
Mouse	*Kit^W/Wv^*	Abdominal aortic banding	↓ perivascular fibrosis	LV	[29]
Mouse	*Kit^W/Wv^*	Transaortic constriction	↓ fibrosis	Atria	[26]
Mouse	*Kit^W/W-sh^*	TNF-α overexpression	↓ fibrosis, ↓ diastolic dysfunction	LV	[30]
Rat	*Ws/Ws*	Hyperhomocysteinemia	↑ fibrosis	LV	[31]
Rat	*Ws/Ws*	Radiation	↑ fibrosis	LV	[32]
Targeting Proteases					
Canine	Chymase inhibitor (SUNC8257)	Pacing-induced heart failure	↓ collagen I and III mRNA, ↓ fibrosis	LV	[33]
Rat	Chymase inhibitor (NK3201)	Myocardial infarction	↓ collagen I and III mRNA, ↓ fibrosis, ↓ E/A	LV	[34]
Mouse	Chymase inhibitor (NK3201)	Intermittent hypoxia	↓ perivascular fibrosis	LV	[35]
Rat	PAR-2 antagonist (FSLLRY, tryptase)	Hypertension	↓ fibrosis	LV	[36]
Mouse	*H2R^−/−^*	Transaortic constriction	↓ fibrosis, ↑ systolic function	LV	[37]

LV = Left ventricle, RV = Right ventricle, PAR-2 = Protease activated receptor-24. MC products.

## Abbreviations

MC	Mast cell
MI	Myocardial Infarction
RV	Right Ventricle
LV	Left Ventricle
SHR	Spontaneously Hypertensive Rat
ECM	Extracellular Matrix
Sl^d^	Steel Dickie
ACE	Angiotensin Converting Enzyme
PAR-2	Protease Activated Receptor 2
H1R	Histamine Receptor 1
H2R	Histamine Receptor 2
H3R	Histamine Receptor 3
H4R	Histamine Receptor 4
RAS	Renin Angiotensin System
HMC-1	Human Mast Cell Line 1
MMP	Matrix Metalloproteinase
TNFRI	Tumor Necrosis Factor Receptor I
TNFRII	Tumor Necrosis Factor Receptor II
C5a	Complement Factor 5a
Ovx	Ovariectomised
